# The impact of transition from paper to electronic death records on garbage codes in the Norwegian Cause of Death Registry 2018–2023

**DOI:** 10.1186/s12889-026-28607-3

**Published:** 2026-08-03

**Authors:** Ingeborg Forthun, Christian Lycke Ellingsen, Carl Michael Baravelli, Kari Anne Sveen, Anne Gro Pedersen, Pétur Benedikt Júlíusson, Ann Kristin Skrindo Knudsen, Marianne Strøm

**Affiliations:** 1 Department of Disease Burden, Norwegian Institute of Public Health, Bergen, Norway; 2 Department of Pathology, Stavanger University Hospital, Stavanger, Norway; 3 Department of Global Public Health and Primary Care, University of Bergen, Bergen, Norway; 4 Department of Health Registry Research and Development, Norwegian Institute of Public Health, Oslo, Norway; 5 Department for Health Data and Collection, Norwegian Institute of Public Health, Oslo, Norway; 6 Department of Clinical Science, University of Bergen, Bergen, Norway; 7 Children and Youth Clinic, Haukeland University Hospital, Bergen, Norway

**Keywords:** Garbage codes, Cause of death, Data quality, Registry-data, Norway

## Abstract

**Background:**

In 2018, electronic death certification was gradually introduced in the Norwegian Cause of Death Registry (NCoDR). Electronic certification was made mandatory from 2022. This may have affected the use and pattern of ill-defined death codes, so-called “garbage codes”, reported as the underlying cause of death. This study aimed to assess whether the transition from paper to electronic certification changed the magnitude and pattern of garbage codes in NCoDR.

**Methods:**

The analyses included all deaths with a registered underlying cause of death in the period 2018–2023 (*n* = 248 225). A garbage code was defined according to the definition used by the Global Burden of Disease study. Trends and patterns of garbage codes were assessed and its association with type of certificate (paper/electronic) estimated using a modified Poisson regression model estimating relative risks with 95% confidence interval (CI) adjusting for sex, age at death and place of death.

**Results:**

In the complete study period, 22.6% of the deaths were registered with a garbage code as the underlying cause of death. The probability of a garbage code was 21% lower in electronic compared to paper certificates (adjusted relative risk 0.79, 95% CI 0.78–0.80). This difference was stable over time and led to an overall decrease in the proportion of garbage codes with increasing use of electronic certifications. The decrease in garbage codes over time was mainly driven by more specific coding. The five most common garbage codes were I50 (Heart failure), J18 (Pneumonia, organism unspecified), I64 (Unspecified stroke), R96 (Other sudden death, cause unknown) and X59 (Accidental exposure to unspecified factor), accounting for more than one third of the garbage codes in the study period. The proportion of all of these, except for I50, were significantly lower in electronic compared to paper certificates, most notably so for J18 and I64.

**Conclusion:**

The transition from paper to electronic certification led to an improvement in cause of death registration through a reduction in garbage codes, although garbage codes remain a considerable problem. An electronic system can provide guidance and feedback to the certifying physician, thereby providing opportunities for further improvement in data quality.

**Supplementary Information:**

The online version contains supplementary material available at https://doi.org/10.1186/s12889-026-28607-3.

## Background

High quality data on the underlying cause of death (UCoD) is essential for monitoring population health, for policy development and research. The UCoD is defined by the World Health Organization (WHO) as “the disease or injury which initiated the train of morbid events leading directly to death, or the circumstances of the accidents or violence which produced the fatal injury” [1]. As the UCoD gives the most information about etiology it is viewed as the most important in terms of prevention of premature mortality. Although WHO provides guidelines and rules for selecting the correct UCoD, registration of a non-informative UCoD can occur due to lack of information about the chain of events or by faults in the certification and coding process [2].

In the Global Burden of Disease Study (GBD) the term “garbage code” is used to describe UCoD that are not specific enough, are an immediate or intermediate cause of death, or an impossible cause of death [3]. The GBD operates with four classes of garbage codes dependent on its policy implications [4]. Class 1 garbage codes are causes in which the true UCoD might belong to any of the three broad cause groups: communicable diseases, non-communicable diseases, or injuries (e.g. R96.0 Sudden death). Class 2 garbage codes are causes that can belong to one, or at most two of the mentioned broad cause groups (e.g. X59.0 Accidental exposure to unspecified factor). Class 3 are causes for which the true UCoD is likely within the same ICD chapter as the garbage code reported (e.g. I51.9 Heart disease, unspecified), while class 4 are causes in which the true UCoD is likely to be confined to a single disease or injury category (e.g. J18.9 Pneumonia, unspecified). Together, class 1 and 2 constitute what are called major garbage codes, while class 3 and 4 are minor garbage codes (see Supplementary material, Sect. 1.1, for further details).

Garbage codes distort true mortality patterns and reduce the usefulness of cause-of-death data for planning and evaluating preventive measures [3, 5]. A high proportion of such codes reduces the number of deaths in the disease group where the true cause belongs. Consequently, a reduction in garbage codes can even create the illusion of false increases or decreases in valid causes of death due to previous under- or overreporting of specific diagnoses. The GBD redistributes all garbage codes to other valid UCoD which in the case of Norway, as for most other countries, results in shifts in ranking of the 20 leading causes of death (see Supplementary material in Johnson, Cunningham et al. 2021). For example, for Norway, death due to cardiovascular disease is ranked higher and death due to cancers lower after garbage code redistribution. However, the redistribution process is associated with uncertainty [6, 7]. Therefore, it is essential to maintain a continuous focus on improving coding practices.

In the period 1996–2019, a total of 29.0% of all deaths in the Norwegian Cause of Death Registry (NCoDR) had a garbage code as the UCoD; 14.1% with a major and 15.0% with a minor garbage code [2]. Over this study period, a decrease in the proportion of minor garbage codes was found, while the proportion of major garbage codes fluctuated over time with an increasing tendency overall. The strongest predictors of a garbage code were older age and place of death outside of hospital. In 36% of the garbage coded deaths, there were other, non-garbage codes mentioned on the death certificate. Among the Nordic countries, Denmark and Sweden have a similar proportion of garbage codes in their national cause of death registries as Norway, while Finland has only about 10% garbage codes [3]. This suggests that measures can be taken to reduce the proportion of garbage codes in the registry.

From 2018, electronic certification was gradually introduced at the NCoDR with the aim to replace paper reports [8]. It was not compulsory until January 2022 but was strongly recommended during the COVID-19 pandemic (starting in March 2020). With the introduction of electronic death certification, an online guidance document was published, and trained personnel from the NCoDR provided courses in each health region and on request. There is no direct linkage between the electronic patient record and the electronic death certificate, and the structure of the electronic certification resembles that of the paper version (see Supplementary material, Sect. 1.2 for further details). There are two parts in the cause of death registration in which part I contains the chain of events leading to death. The physician first registers the immediate cause (a) and then goes stepwise back to the UCoD. Part II contains the contributing causes. One important change from the paper-based to the electronic certificate, was the expansion of the chain of events in part I with one more step, from a-c to a-d, according to recommendations from the WHO. The process begins with the physician manually entering a diagnosis or ICD-10 code, which then triggers a drop-down menu with suggested causes with corresponding ICD-10 codes. Only one cause can be registered at each step. If needed, the physician can add causes manually in a free-text field. The causes included in the free-text field are reviewed and manually registered by the coders at the NCoDR. After completing the chain of events leading to death, the physician is prompted to provide a more specific diagnosis if only an ill-defined code has been entered (as defined by the WHO list; see Supplementary material, Sect. 1.3). As with the paper-based system, the reported string of codes is automatically processed by the coding system Iris [9] based on the Automatic Classification of Medical Entities software (ACME) [10] which selects the UCoD based on the rules provided by the WHO [1]. Certificates that are more complex (e.g. deaths examined by autopsy, deaths from external causes, deaths with codes in free-text-fields, deaths among children less than 1 year of age) are reviewed by specially trained staff from the NCoDR before they are recorded in the register. In about 3% of cases, the staff may also request additional information about the death by contacting the certifying physician (e.g. death certificates flagged by the Iris coding system, and deaths among persons under 80 years old with an ill-defined UCoD according to WHO classification).

The transition from paper to electronic certification may have affected the type of codes registered and the data quality. Therefore, the aim of the current study was to assess whether the magnitude and pattern of garbage codes used as the UCoD changed with the introduction of electronic certification in the Norwegian Cause of Death registry. And further, whether the proportion of garbage coded deaths with other informative (non-garbage codes) elsewhere on the certificate changed. We hypothesized that the introduction of electronic certification led to a reduction in the use of garbage codes as the UCoD with a potential effect on the type of garbage codes used.

## Methods

We used data on all deaths among Norwegian residents occurring from January 1st, 2018, to 31st of December 2023 with a registered UCoD (data last updated in September 2024). The classification of garbage codes was based on the GBD2021 study [11]. A mapping from ICD-10 codes to garbage codes was derived from the GBD2021 capstone paper on causes of death and life expectancy. Garbage codes in GBD class 1 and 2 were defined as major garbage codes, and class 3 and 4 as minor (Supplementary Table 1, Sect. 1.1).

Descriptive trends in garbage codes, in total and by type of certificate were assessed. In addition, regression analyses were run with the probability of a garbage code reported as the UCoD as the dependent variable and type of death certificate (with paper certification as the reference level) as the independent variable. Both crude and adjusted models were estimated adjusting for potential confounders identified in previous work [2, 12]: age at death (< 1, 1–19, 20–49, 50–79 (reference), 80–89, 90 + years), sex (male, female (reference)) and place of death (hospital (reference), nursing home, at home, other known (e.g. public place, at sea)) in main model. In addition, a model adjusting for other potential confounders with available information in the registry was estimated. This included, in addition to age, sex and place of death, whether an autopsy was performed (no autopsy (reference), non-forensic, forensic), centrality index (high (reference), medium, low), number of ICD-10 codes on the registration form (continuous variable, included as a quadratic term to allow for an observed non-linear association) and whether additional information had been requested by the registry (yes, no (reference)). The centrality index was based on the Standard Classification of Municipalities that measures a municipality’s geographical position seen in relation to service functions and amount of workplaces [13]. In the present study this pertains to the municipality where the death occurred. Deaths in which place of death, autopsy or centrality index was not known were excluded from the adjusted analyses. To assess potential differences in garbage code use over time for type of certificate (electronic/paper) and age, models including an interaction term with year of death (included as a categorical variable) were also run. In these analyses, the two youngest age groups were collapsed due to very few garbage codes in the youngest age group (< 1 year old).

In all regression analyses, a modified Poisson regression model with robust standard errors was used to estimate crude and adjusted relative risks (RR) with 95% confidence intervals (CI). This model approach is recommended as an alternative to the more conventional binomial approach in cases where one wants to estimate RR, but the log binomial model fails to converge [14, 15]. A likelihood ratio test comparing the adjusted model with and without an interaction term was used to test for interaction.

To explore differences in patterns of garbage codes by type of certificate (paper or electronic), the five most common garbage codes were listed and their association with type of certificates assessed. For this analysis, garbage codes that only differed in the fourth character of the ICD-10 code were grouped together. Lastly, the proportion of certificates with a major or minor garbage code that had a non-garbage code listed somewhere else on the certificate was calculated.

For all statistical analyses, we used R (version 4.3.0). The modified Poisson regression model with robust standard errors was estimated using the lmtest and sandwich packages in R.

## Results

### Overview of data material

All deaths in the period 2018–2023, in total *n* = 253 343, were included. In 5117 of these deaths, the death certificate was missing (2.0%). In addition, one deceased with a death certificate had no registered UCoD. These deaths were excluded from the analyses, resulting in a final study sample of *n* = 248 225.

The number of deaths in Norway fluctuated around 40 000 each year but was higher the last two years (2022 and 2023). The study sample included 121 742 men (49.0%) and 126 483 women (51.0%) (Table 1). In the complete study period, 57.6% of the deaths occurred in persons 80 years old or above, but this varied by sex; 49.4% in males and 65.5% in females. Most deaths occurred either at a nursing home (53.6%) or at a hospital (28.5%), while an autopsy was conducted in 8.4% of all deaths in the study period (Table 1). In 2.9% of the deaths, additional information was requested by the registry (Table 1), but this proportion varied by type of certification with 2.2% in deaths reported on paper and 3.4% in deaths certified electronically.

**Table 1:** Number and column percentage of deaths by potential confounders, Norway 2018–2023

		*N*	Column %
Sex	Male	121 742	49.0
	Female	126 483	51.0
Age at death	0	612	0.2
	1–19	917	0.4
	20–49	8 554	3.4
	50–79	95 173	38.3
	80–89	81 176	32.7
	90+	61 793	24.9
Place of death	At home	35 564	14.3
	Hospital	70 805	28.5
	Nursing home	133 128	53.6
	Other known	7 227	2.9
	Unknown	1 501	0.6
Autopsy	No autopsy	227 404	91.6
	Non-forensic	7 533	3.0
	Forensic	13 287	5.4
	Unknown	1	0.0
Centrality^a^	Low	27 550	11.1
	Medium	101 945	41.1
	High	106 462	42.9
	Missing data	12 268	4.9
Additional information requested			
	No	241 102	97.1
	Yes	7 123	2.9
Number of diagnoses		Median	IQR
		3	2–4

^a^The centrality index pertains to the geographical position of the municipality where the death occurred. The index is based on the Standard Classification of Municipalities that measures a municipality’s geographical position seen in relation to service functions and amount of workplaces [13]

### Descriptive results

**Fig. 1:**
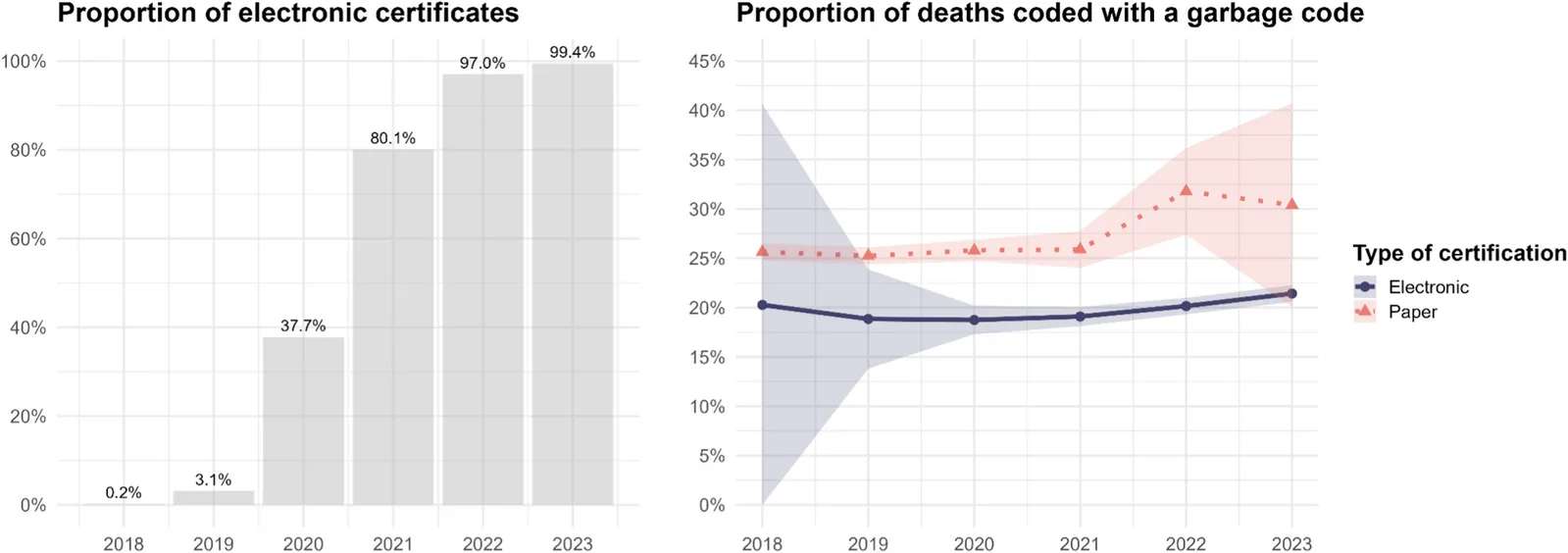
Proportion of deaths certified electronically (panel left), and proportion of deaths with a garbage code in paper and electronic certificates by year with 95% confidence interval (shaded area) (panel right), 2018–2023

In 2018, the year when electronic certification was introduced, only 74 deaths were certified electronically, but this gradually increased over time (Fig. 1, left panel). In 2022, 43 859 deaths (97.0%) and in 2023, 42 869 (99.4%), were certified electronically. Overall, from 2018 to 2023, 54.7% of deaths were certified electronically and 22.6% (95% CI 22.5–22.8%) reported with a garbage code. The percentage of garbage was lower in electronic certificates (20.1%, 95% CI 19.9–20.3%) than in paper certificates (25.6%, 95% CI 25.4–25.9%) (Table 2). The proportion of garbage codes in electronically certified deaths remained stable over time apart from a slight increase observed in 2022 and 2023 (Fig. 1, right panel).

**Table 2:** Crude and adjusted relative risk (RR) of garbage code use by type of death certificate, Norway 2018–2023

		Percentage garbage codes (95% CI)	Crude RR (95% CI) Model 1	Adjusted RR^1^ (95% CI) Model 2	Adjusted RR^2^ (95% CI) Model 3
Type of death certificate	Paper	25.6 (25.4–25.9)	1.00 (ref.)	1.00 (ref.)	1.00 (ref.)
	Electronic	20.1 (19.9–20.3)	0.79 (0.77–0.80)	0.79 (0.78–0.80)	0.77 (0.76–0.78)

^1^Adjusted for age at death, sex and place of death

^2^Adjusted for age at death, sex, place of death, autopsy, centrality index, number of diagnoses on certificate and whether additional information had been requested by the registry

The gradual increase in the use of electronic certificates corresponded with a decrease in the proportion of garbage codes in total. In 2018, the proportion of garbage codes was 25.6% (95% CI 25.2–26.1%), 14.2% major (class 1–2, 95% CI 13.9–14.6%) and 11.4% minor (class 3–4, 95% CI 11.1–11.7%). In 2023, this had decreased to 21.5% garbage codes in total (95% CI 21.1–21.9%), 13.7% major (95% CI 13.3–14.0%) and 7.8% minor (95% CI 7.6–8.1%). Hence, the decrease in garbage codes overall was mainly driven by a decrease in the use of minor garbage codes, particularly the use of class 4 garbage codes (Supplementary Fig. 3, Sect.  2). A decrease in proportion of class 2 garbage codes was also seen, but this was offset by a slight increase in class 1 garbage codes (Supplementary Fig. 3, Sect.  2).

### Results from regression analyses

A log-binominal regression model was estimated to explore the association between electronic certification and the probability of garbage code reported as the UCoD when adjusting for potential confounders. When adjusting for age, sex and place of death, electronic certification was associated with a 21% reduction in the probability of garbage code reported as the UCoD (adjusted RR 0.79, 95% CI 0.78–0.80) (Table 2, Model 2). Further adjustment for all potential confounders gave a very similar result (Table 2, Model 3). Crude and adjusted results for all potential confounders are displayed in Supplementary Table 3 (Sect. 2 of Supplementary material).

**Fig. 2:**
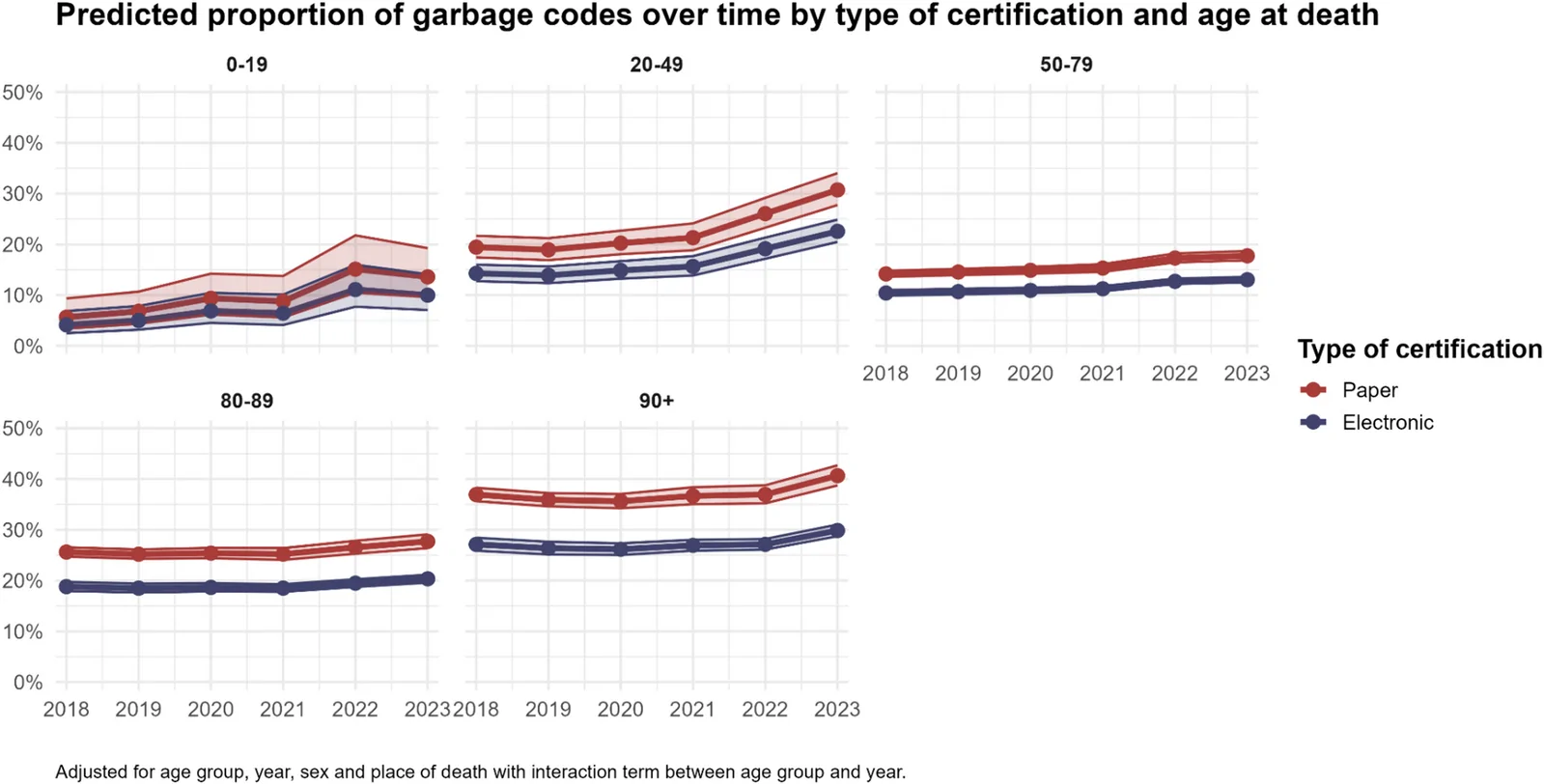
Predicted proportion of garbage codes by type of certificate and age group adjusting for age of death, sex, and place of death, with interaction term between age at death and year of death, when setting place of death and sex equal to the reference group (“Hospital” and “Female”) to simplify the graphical representation

No indication of an interaction effect between type of certification and year of death was found (p-value = 0.51). The association between age groups and garbage codes, however, differed over time as shown in Fig. 2 (*p*-value < 0.01). A slight increase in garbage codes over time was seen for age group 0–19, in addition to an increase the last two years for age group 20–49 (Fig. 2). However, the increase for age group 20–49 was less apparent when not defining X40-X44 (Accidental poisonings due to drugs and substances of abuse) as garbage codes (Supplementary Fig. 4, Sect.  2). These are considered garbage codes by the GBD but not as an ill-defined cause of death by the WHO.

### Patterns of garbage codes

In the complete study period, the five most common garbage codes were I50 (Heart failure), J18 (Pneumonia, organism unspecified), I64 (Unspecified stroke), R96 (Other sudden death, cause unknown), and X59 (Accidental exposure to unspecified factor), together accounting for 35.5% of the garbage codes in total (42.9% for certificates on paper and 27.7% for electronic certificates). The pattern of garbage codes was similar in paper and electronic certificates (Supplementary Table 4, Sect.  2), however, for all the ICD-10 codes mentioned, the proportion of garbage-coded deaths with the specific garbage code was statistically significantly higher in deaths reported on paper, most notably so for J18 (Pneumonia, organism unspecified) and R96 (Other sudden death, cause unknown) (Table 3). The only exception to this was I50 (Heart failure) in which the proportion of garbage-coded deaths with this code was about the same in deaths certified electronically compared to on paper. During the study period, there was an increase in deaths reported with R99 (Ill-defined and unknown cause of mortality) and A49 (Bacterial infection of unspecified site) as the UCoD (Supplementary Fig. 5, Sect. 2). The decrease in proportion of minor garbage codes seen over time, as mentioned above, was largely attributed to a decrease in the use of the two most common minor garbage codes; J18 (Pneumonia organism unspecified) and I64 (Stroke, not specified as hemorrhage or infarction) (Supplementary Fig. 5, Sect. 2).

**Table 3:** The five most common garbage codes in Norway 2018–2023 in all deaths with a garbage code, with number, proportion and absolute difference between deaths certified on paper and deaths certified electronically

Diagnostic code (major/minor)	All deaths	Paper	Electronic	
	*N* (% of garbage code)	*N* (% of garbage codes)	*N* (% of garbage codes)	Absolute difference in proportion (%) (95% CI)
All certificates with a garbage code	56 164 (100.0)	28 820 (100.0)	27 344 (100.0)	
I50 Heart failure (major)	6574 (11.7)	3273 (11.4)	3301 (12.1)	-0.7 (-1.25- -0.18)
J18 Pneumonia, organism unspecified (minor)	4560 (8.1)	3605 (12.5)	955 (3.5)	9.0 (8.6–9.5)
I64 Stroke, not specified as hemorrhage or infarction (minor)	3537 (6.3)	2333 (8.1)	1204 (4.4)	3.7 (3.3–4.1)
R96 Other sudden death, cause unknown (major)	2725 (4.9)	1605 (5.6)	1120 (4.1)	1.5 (1.2–1.8)
X59 Accidental exposure to unspecified factor (major)	2526 (4.5)	1542 (5.4)	984 (3.6)	1.8 (1.4–2.1)

Of the 56 164 deaths reported with a garbage code as the UCoD, 13 721 (24.4%, 95% CI 24.1–24.8) had one or more non-garbage code among the registered codes on the certificate. This proportion varied by type of certification and was lower for deaths certified electronically (21.1, 95% CI 20.6–21.6) than among those certified on paper (27.6%, 95% CI 27.1–28.1).

## Discussion

This study sought to describe whether the magnitude and pattern of garbage codes in the Norwegian Cause of Death Registry changed with the gradual introduction of electronic certification in the years 2018–2023. We found that the proportion of deaths with a garbage code as the UCoD was consistently lower in deaths reported electronically. This difference was consistent and stable over time and led to a decrease in the proportion of garbage codes over time as more and more deaths were reported electronically. The decrease in garbage codes was mainly driven by a decrease in the use of minor garbage codes, in particular garbage codes of class 4. The five most common major garbage codes were I50 (Heart failure), J18 (Pneumonia, organism unspecified), I64 (Unspecified stroke) R96 (Other sudden death, cause unknown) and X59 (Accidental exposure to unspecified factor), together accounting for more than one third of all garbage codes.

In the first year of electronic certification (2018), only 74 of the included deaths were certified electronically. Electronic certification was planned to be made mandatory from 1st of January 2022. However, when the Covid-19 pandemic hit Norway in March 2020, all healthcare institutions and municipalities were strongly encouraged to report deaths electronically for timely surveillance of death [8]. This probably gave a much more rapid increase in use of electronic certification than what would have happened had there not been a pandemic. As the transition from paper to electronic certification overlaps with the pandemic, it is difficult to assess the real causal effect of electronic certification on use and pattern of garbage codes. The pandemic may have had an independent effect on coding practice due to an increased focus on identifying the cause of death, maybe in particular in cases of respiratory infections [16]. This could for example help explain the decrease in the use of J18 (Pneumonia, organism unspecified), which corresponded to an increase in J15.9 (Unspecified bacterial pneumonia), also a garbage code, but a more specific one resulting in increased data quality. Although it should be noted that a decrease in J18 also was seen prior to the pandemic [2]. In addition, the presentation and ordering of ICD-codes in the drop-down menu might also be a factor.

Several mechanisms may underlie the lower probability of garbage codes in electronic certificates, in addition to the potential effect of the pandemic as discussed above. For example, additional training was provided when electronic certification was introduced, both across health regions and upon request. Real-time feedback in which a warning message is shown if reporting only an ill-defined code was also introduced from 2020. The absence of any interaction effect, with a further reduction in the probability of a garbage code in electronic certified deaths over time, may suggest a selection effect. Electronic certification was introduced gradually, initially targeting institutions with high numbers of deaths. Those who adopted to electronic registration early were likely the most motivated and experienced in certifying deaths.

Although many countries still use paper-based systems, there is a trend towards introducing electronic systems [17]. A study that assessed the transition from paper to electronic certification in the period 2012–2014 in Portugal found improvements in data quality, although a measure of garbage code use was not included in this study [18]. A similar study in France, following the introduction of electronic death certification in 2007, also reported higher data quality, in the form of more complete information, in electronic compared to paper certificates [19]. Pilot studies of online certification in Germany and Perú have reported declines in proportion of ill-defined UCoD [20, 21].

In the present study, the lowest overall proportion of garbage codes was found in 2021 followed by a slight increase in major garbage codes in 2022 and 2023. Although Norway, in a global comparison, had few excess deaths during the pandemic [11], excess mortality across all age groups was seen both in 2022 and 2023 [22]. It is likely that the increase in number of deaths had a negative effect on the ability to thoroughly assess the chain of events leading to death, both for the physician and the registry. In 2023, excess mortality was also seen among younger age groups, in particular for traffic and drug-related deaths [23], deaths that normally are associated with more garbage codes. The increase in garbage codes in 2022 and 2023 for the age group 20–49 was less apparent when defining X40-X44 (accidental poisonings due to drugs and substances of abuse) as non-garbage codes. These codes are considered garbage by the GBD but are not listed as ill-defined causes of death by the WHO [24]. The increase in garbage codes in 2023 could also be partly due to late arriving reports from forensic autopsies, in which it can take up to one year before the UCoD is determined and reported.

The pattern in the most used major and minor garbage codes was similar for paper and electronic certificates. The largest difference, and the main driver of the decrease in garbage codes over time, was a rapid decrease in J18 (Pneumonia organism unspecified) and I64 (Stroke, not specified as hemorrhage or infarction). Both of these are defined as class 4 garbage codes by the GBD; diagnoses in which the true UCoD likely is within the same disease or injury category and the implication of of garbage codes is limited [4]. It is likely that the electronic system helps the certifying doctor to be more specific by presenting several alternative diagnoses in the drop-down menu, although the drop-down menu may also introduce systematic errors [25]. In addition, it is important to note that more specific coding does not necessarily imply more correct coding. Although the GBD distinguishes four levels of garbage codes based on their policy implications, minor codes may sometimes merely act as proxies for more severe garbage codes at levels 1–2. Apart from a slight decrease in class 2 garbage codes over time, the proportion of major garbage codes overall (class 1–2) was stable. The observed increase in deaths reported with R99 (Ill-defined and unknown cause of mortality) during the study period coincides with a decline in deaths missing either a death certificate or an UCoD [26]. With the implementation of electronic certification, both administrative and medical death registration are completed simultaneously. As documentation completeness improves, some cases that previously lacked a certificate or UCoD may now be reported with R99, indicating that while the certificate is submitted, the underlying cause remains unspecified.

The proportion of deaths in which more information had been requested by the registry was higher in electronically certified deaths. The electronic system facilitates for direct electronic communication with the certifying doctor when additional information is needed, which very likely also contributes to increased data quality. About 25% of the certificates with a garbage code had another non-garbage code on the certificate which is lower than reported in a previous publication with data from the NCoDR from 1996 to 2019 (36.0%, 95% CI 35.9–36.2%) [2]. In the present study, this percentage was much lower for electronic than for paper certificates indicating that garbage coding in the electronic system to a greater degree is due to insufficient information rather than a fault in certification. Other valid codes on the certificate could give valuable information on co-morbidity, but not necessarily on the real UCoD. Further, the registration of the underlying cause of death in the NCoDR follows the rules provided by the WHO [24] which is shorter than the list of garbage codes in the GBD.

### Methodological considerations and limitations

This is a large study including all deaths in Norway in the period 2018–2023 with a registered UCoD (98.0%). The classification system ICD-10 has been used throughout the study period. However, differences in coding practices over time may have affected our results, with the COVID-19 pandemic as a potential important driver for both short-term and long-term change as discussed above.

Although the use of the GBD garbage code list makes it possible to compare the study results with other previous studies conducted in Norway, the Nordic region and internationally, it is not in line with the practice at the registry. The registration of the UCoD in the NCoDR follows the rules provided by the WHO [24]. The list of ill-defined codes in the WHO is shorter and differs from the GBD list of garbage codes. The latter is generated by experts specializing in causes of death at the Institute of Health Metrics and Evaluation and reviewed and approved by the GBD Scientific Council [11]. The list of garbage codes and the classification of garbage codes across levels 1–4 have changed over time with each iteration of the GBD (see Supplementary appendix 1 in Naghavi et al. 2024). As a result of the discrepancy between GBD and WHO, the results would have been different had we used the list of ill-defined causes according to the WHO, in particular for the younger age groups.

About 13% of the certificates were sent in more than once both on paper and electronically. Regardless, these were coded as electronic certificates. This could result in a misclassification if for example the final underlying cause of death was more often registered on paper.

### Implications of results

Although registration of a garbage code as the UCoD sometimes is unavoidable due to lack of information about the deceased and chain of events leading to death, many of the registered garbage codes are probably due to a fault in certification and could have been avoided. Norway, as Denmark, Iceland, and Sweden, has a much higher proportion of garbage codes than neighboring country Finland where only about 10% of deaths are reported with a garbage code [3]. This difference across the five Nordic countries, all with robust social security systems and a universal, publicly financed health care systems, suggests a potential for further improvement of the quality of the cause of death data. The case of Finland suggests that there is room for improvement in Norway also when using the shorter list of ill-defined codes from WHO as a benchmark. Finland has a much higher rate of forensic autopsies in deaths with unknown or ill-defined cause of death compared to the other Nordic countries, which has been suggested to explain some of the difference in garbage coding [27]. In addition, all death certificates in Finland are reviewed by medical examiners. If a certificate includes a non-informative UCoD, it is returned to the certifying physician with written instructions for correction. Physicians can also call a dedicated phone number for guidance on completing the certificate [28]. The experience from Finland suggests that a combination of training, guidance, and enhanced quality assurance can further reduce the proportion of ill-defined UCoD, as supported in international literature [20, 25, 29]. Manually reviewing all death certificates is resource intensive. However, the electronic system makes it possible to provide guidance and feedback to the certifying physicians. Although some real-time feedback has already been introduced – by asking them to be more specific when the only cause reported on the certificate is an ill-defined code (according to the WHO list, see supplementary Table 2, Sect. 1.3) – more guidance should be incorporated and preferably, its effect monitored. Overall, quite few ICD-10 codes contributed to a large proportion of the garbage coded deaths. Hence, targeted feedback on these specific codes could help increase data quality further. There is currently no direct link between electronic death certificates and patient records which could help further reduce the proportion of garbage codes [30].

An assessment of the association between predictors such as age, sex, and place of death with the probability and patterns of garbage codes in electronic certificates—which are now the standard—was beyond the scope of this study, but could provide valuable insights for further improving data quality. Further, information about the sex, age, position, and year of experience of the certifying doctor can provide more information on how to improve the quality of the cause of death data. At present, this information is not registered in the system. As the certificate includes the whole chain of events leading to death and not only the UCoD, patterns of multiple causes of deaths could be used to identify potential patterns in frequently selected garbage codes [31]. In addition, as data from the NCoDR can be linked to other national registries, information on the death certificate can be compared with patient records to identify patterns such as under-reporting of certain conditions. Furthermore, a mixed-method approach—where two physicians certify the same death and their agreement is assessed, combined with focus group discussions to explore challenges in death certification—could yield insights to further improve data quality.

Social inequalities in premature mortality are a major public health problem in Norway [32]. High quality cause of death data is required to address this issue with targeted and effective public health policies [33]. A recent Norwegian study on the report of heart failure as the UCoD (defined as a garbage code both by the WHO and the GBD) alludes to an inverse educational gradient in garbage codes [34]. An educational gradient in garbage codes will have a negative effect on our ability to address social inequalities in premature mortality and should be further explored across all garbage codes.

## Conclusion

The present study indicates that the transition to electronic certification improved the quality of the cause of death data, likely through several different mechanism. Even so, garbage codes are still a considerable problem in the Norwegian cause of death statistics. Garbage codes distort statistics, resulting in both an overestimation of the prevalence of some UCoD, and the underestimation of others [5]. As a result, the cause of death statistics does not reflect the true health status of the population. The present study shows how there is room for further improvement and offers guidance on how to achieve them.

## Supplementary Information

Supplementary Material 1.

## Data Availability

The data that support the findings of this study are available from the Norwegian Cause of Death Registry, but restrictions apply to the availability of these data, which were used under license for the current study, and so are not publicly available. Data are however available from the authors upon reasonable request and with permission of the Norwegian Cause of Death Registry.

## Abbreviations

WHO: World Health Organization
UCoD: Underlying cause of death
NCoDR: Norwegian Cause of Death Registry
GBD: Global Burden of Disease
