# Supplementary material for: The impact of transition from paper to electronic death records on garbage codes in the Norwegian Cause of Death Registry 2018–2023
Source: BMC Public Health. 2026 Aug 3;26:3131. doi: 10.1186/s12889-026-28607-3 (PMC13721235; doi:10.1186/s12889-026-28607-3)
Supplement: Supplementary file 1 [file 12889_2026_28607_MOESM1_ESM.docx]

**Supplementary material**

**Section 1: Background information**

**Section 1.1 List of garbage codes in the Global Burden of Disease (GBD)**

The classification of garbage codes used in the present study is based on the Global Burden of Disease 2021 study [1]. In the GBD, the garbage codes are categorized in four classes dependent on their implication on public health. This refers to how many other ICD-10 codes the specific garbage code may mask. In the GBD, all causes of death are organized in a hierarchical structure with four levels. At the first level there are three categories: (1) communicable, maternal, neonatal, and nutritional causes, (2) noncommunicable diseases, and (3) injuries. At level 2, there are 22 categories within each of the three categories at level 1 which are further divided into level 3 and level 4 causes. Class 1 garbage codes include ICD-10 codes in which the true UCoD might belong to any of the three broad cause groups at level 1 in the disease hierarchy, while for class 2 it is assumed that the true UCoD are causes that belong to one, or at most two specific groups of the three mentioned categories at level 1. Example of class 1 garbage codes are R96.0 Sudden death, R99.0 Ill-defined and unknown cause of death and I50.0 Heart failure, while X59 Accidental exposure to unspecified factor and Y86 Sequale of othe accident are examples of class 2 garbage codes. Class 3 are causes for which the true UCoD is likely within the same ICD chapter as the garbage code reported. Examples are I51.9 Heart disease, unspecified and A31.0 Pulmonary mycobacterial infection. Class 4 garbage codes are causes in which the true UCoD is likely within the same disease or injury category, such as J18.9 Pneumomia, unspecified and I64.0 Stroke, not specified as hemorrhage or infarction.

Supplementary table 1: List of garbage codes according to the Global Burden of Disease study (GBD2021), by class 1-4.

| **Class 1** | |
| --- | --- |
| **Chapter in ICD-10** |  |
| I Certain infectious and parasitic diseases | A40-A41.9, A48.0, A48.3, A49.0-A49.1, A59-A59.9, A71-A71.9, A74.0, B07-B07.9, B30-B30.9, B35-B36.9, B85-B85.4, B87-B88.9, B94.0 |
| III Diseases of the blood and bood-forming organs and certain disorders involving the immune mechanism | D50-D50.0, D50.9, D62-D63.0, D63.8-D64, D64.1-D65.9, D68, D69.9 |
| IV Endocrine, nutritional and metabolic diseases | E15-E16, E50-E50.9, E64.1, E85.3-E87.6, E87.8-E87.9 |
| V Mental and behavioural disorders | F06.2-F06.4, F07.2, F09-F09.9, F19-F23.9, F25-F49, F51-F99.0 |
| VI Diseases of the nervous system | G06-G08.0, G32-G32.8, G43-G44.2, G44.4-G44.8, G47-G47.2, G47.4-G47.9, G50-G60.9, G62-G62.0, G62.2-G65.2, G80-G83.9, G89-G89.4, G91-G91.2, G91.4-G93, G93.1-G93.2, G93.4-G93.6, G94.0-G94.8, G99 |
| VII Diseases of the eye and adnexa | H00- H05, H05.2-H59 |
| VIII Diseases of the ear and mastoid process | H60-H69.9, H71-H99 |
| IX Diseases of the circulatory system | I26-I26.9, I31.2-I31.4, I46-I46.9, I50.0-I50.4, I76, I95-I95.1, I95.8-I95.9 |
| X Disease of the respiratory system | J69-J69.9, J80-J80.9, J81.0, J85-J85.3, J86-J86.9, J90-J90.0, J93-J93.1, J93.8-J94.9, J96-J96.9, J98.1-J98.3 |
| XI Diseases of the digestive system | K00-K19, K30, K65-K66.1, K66.9, K68.1-K68.9, K71-K71.6, K71.8-K72.9, K75.0 |
| XII Diseases of the skin and subcutaneous tissue | L20-L30.9, L40-L50.9, L52-L54.8, L56-L56.2, L56.4-L56.5, L57-L57.9, L59-L68.9, L70-L76.8, L80-L87.9, L90-L92.9, L94-L96, L98.5-L99.8 |
| XIII Diseases of the musculoskeletal system and connective tissue | M04, M10-M12.0,  M12.2-M29, M37-M39, M43.2-M49, M49.2-M64, M65.1-M71, M71.2-M72.4, M72.8-M73, M73.8-M79.9, M83-M86.2, M86.5-M86.9, M87.2-M87.9, M89.1-M89.4, M90-M99.9 |
| XIV Disease of the genitourinary system | N17-N17.9, N19-N19.9, N32.1-N32.2, N32.8-N33.8, N35-N35.9, N37-N37.8, N39.3-N39.8, N42-N43.4, N44.1-N44.8, N46-N48.9, N50-N53.9, N61-N64.9, N82-N82.9, N91-N91.5, N95, N95.1-N95.9, N97-N97.9 |
| XVIII Symptoms, signs and abnormal clinical and laboratory findings, not elsewhere classified | R02-R02.9, R03.1-R04, R04.1-R04.9, R07.0, R08-R12.0, R14-R19.6, R19.8-R23, R23.1-R30.9, R32-R50.1, R50.8-R57.9, R58.0-R72.9, R74-R78, R78.6-R94.8, R96-R99.9 |
| XX External causes of morbidity and mortality | X40-X44.9, X46-X46.9, X49-X49.9, Y10-Y14.9, Y16-Y19.9 |
| XXI Factors influencing health status and contact with health services | Z00-Z15.8, Z17-unsp. |
| XXII Codes for special purposes | U05, U08-U81, U89.9-U99 |
| **Class 2** | |
| I Certain infectious and parasitic diseases | A14.9, A29-A30.9, A45-A45.9, A47-A48, A48.8-A49, A49.3-A49.9, A61-A62, A72-A73, A76, A97, B08-B09, B11-B14, B28-B29, B31-B32.4, B34-B34.1, B34.3-B34.9, B61-B62, B68-B68.9, B73-B74.2, B76-B76.9, B78-B81.8, B84, B92-B94, B94.8-B94.9, B95.6-B97.1, B97.3, B97.7-B99.9 |
| III Diseases of the blood and bood-forming organs and certain disorders involving the immune mechanism | D59, D59.4, D59.8-D59.9 |
| V Mental and behavioural disorders | F17-F17.9 |
| VI Diseases of the nervous system | G44.3, G91.3, G93.0, G93.3 |
| IX Diseases of the circulatory system | I10-I10.9, I15-I15.9, I27, I27.8-I27.9, I50, I50.8-I50.9, I67.4, I70-I70.1, I70.9, I74-I75.8 |
| X Diseases of the respiratory system | J81, J81.1 |
| XI Diseases of the digestive system | K92.0-K92.2 |
| XIV Diseases of the genitourinary system | N70-N71.9, N73-N74.0, N74.2-N74.8 |
| XVIII Symptoms, signs and abnormal clinical and laboratory findings, not elsewhere classified | R03-R03.0, R04.0, R05-R06.9, R13-R13.9, R23.0, R58 |
| XIX Injury, poisoning and certain other consequences of external causes | S00-T98.3 |
| XX External causes of morbidity and mortality | W47-W48, W63, W71-W72, W76-W76.9, W82, W95-W97, W98, X07, X55-X56, X59-X59.9, Y20-Y34.9, Y86-Y87, Y87.2, Y89, Y89.9-Y99.9 |
| **Class 3** | |
| I Certain infectious and parasitic diseases | A01, A31-A31.9, A42-A44.9, A49.2, A64-A64.0, A99-A99.0, B17, B17.1, B17.8-B17.9, B19-B19.0, B19.2-B19.9, B37-B46.9, B49-B49.9, B55, B55.1-B55.9, B58-B59.9, B89, B94.2, - |
| II Neoplasms | C14-C14.9, C22.9, C26-C29, C35-C36, C39-C39.9, C42, C46-C46.9, C55-C55.9, C57.9, C59, C63.9, C68, C68.9, C74-C74.9, C75.9-C80.9, C83, C83.9, C85.1, C85.9, C87, C94.6, C97-D00.0, D01, D01.4-D02, D02.4-D02.9, D07, D07.3, D07.6-D09, D09.1, D09.7, D09.9-D10, D10.9, D13, D13.9-D14, D14.4, D17-D21.9, D28, D28.9-D29, D29.9-D30, D30.9, D36.0, D36.9-D37.0, D37.6-D38, D38.6-D39.0, D39.7, D39.9-D40, D40.9-D41, D41.9, D44, D44.9, D48, D48.7-D49.1, D49.5, D49.7-D49.9 |
| III Diseases of the blood and blood-forming organs and certain disorders involving the immune mechanism | D54, D75.9, D79-D85, D87-D88, D89.8-D99 |
| IV Endocrine, nutritional and metabolic diseases | E07.8-E08.9, E17-E19, E34.0, E34.9-E35.8, E37-E39, E47-E49., E62, E69, E87.7, E90-E998 |
| V Mental and behavioural disorders | F04-F06.1, F06.5-F07.0, F07.8-F08, F50, F50.8-F50.9 |
| VI Diseases of the nervous system | G09-G09.9, G15-G19, G21, G21.2, G21.4-G22.0, G27-G29, G33-G34, G38-G39., G42, G48-G49, G66-G69, G74-G79, G84-G88, G93.8-G94, G96-G96.9, G98-G98.9 |
| IX Diseases of the circulatory system | I00.0, I03-I04., I14-I14., I16-I19, I29-I29.9, I44-I45.9, I49-I49.9, I51, I51.6-I59, I90-I94, I96-I96.9, I98.4-I98.8, I99-ID5.9 |
| X Diseases of the respiratory system | J02.9, J03.9, J04.3, J06, J06.9, J40-J40.9, J47-J59, J65-J65.0, J71-J79, J81.9, J83, J85.9, J87-J89, J90.9, J93.6, J97-J98.0, J98.4-J99.8 |
| XI Diseases of the digestive system | K21-K21.9, K22.7, K31.9-K34, K39, K47-K49, K53-K54, K63-K63.4, K63.8-K63.9, K69, K70.4-K70.9, K78-K79, K84, K87-K89, K92, K92.9-K93, K96-K99 |
| XII Diseases of the skin and subcutaneous tissue | L06-L07, L09, L15-L19, L31-L39, L69, L77-L79 |
| XIV Diseases of the genitourinary system | N09, N13-N13.5, N13.7-N13.9, N24, N28.8-N28.9, N38, N39.9-N40.9,  N54-N59, N66-N69, N78-N79, N84, N84.2-N86, N88-N90.9, N92-N94.9, N95.0 |
| XV Pregnancy, childbirth and the puerperium | O08-O08.9, O17-O19, O27, O37-O39, O49-O59, O78-O79, O93-O95.9 |
| XVI Certain conditions originating in the perinatal period | P06, P16-P18, P30-P34.2, P40-P49, P62-P69, P73, P79, P82, P85-P89, P96.9-P99.9 |
| XVII Congenital malformations, deformations and chromosomal abnormalities | Q08-Q10.3, Q19, Q29, Q36.0-Q36.9, Q46-Q49, Q88, Q89.9, Q94, Q99.9 |
| XVIII Symptoms, signs and abnormal clinical and laboratory findings, not elsewhere classified | R00-R01.2, R07, R07.1-R07.9, R31-R31.9 |
| **Class 4** | |
| I Certain infectious and parasitic diseases | B16.9, B54-B54.0, B64, B82-B82.9, B83.9, |
| II Neoplasms | C69, C69.9, C91.1, C91.4-C91.5, C91.7-C91.9, C92.7-C92.9, C93.2, C93.5-C93.7, C93.9 |
| IV Endocrine, nutritional and metabolic diseases | E12-E14.9 |
| VI Diseases of the nervous system | G00, G00.9-G02.8, G03.9 |
| IX Diseases of the circulatory system | I37.9, I42-I42.0, I42.9, I51.5, I64-I64.9, I67, I67.8-I68, I68.8-I69, I69.4-I69.9 |
| X Diseases of the respiratory system | J07-J08, J15.9, J17-J19.6, J22-J29, J64-J64.9 |
| XVI Certain conditions originating in the perinatal period | P23, P23.5-P23.9, P37.3-P37.4 |
| XVIII Symptoms, signs and abnormal clinical and laboratory findings, not elsewhere classified | R73-R73.9 |
| XX External causes of morbidity and mortality | V87-V87.1, V87.4-V88.1, V88.4-V89.9, V99-V99.0, X84-X84.9, Y09-Y09.9, Y85-Y85.9 |

**Section 1.2 Structure of the cause of death certificate**

The structure of the paper and electronic certificate when registering the chain of events leading to death is similar. A screenshot of the paper and electronic certificate is included below in Supplementary figure 1 and 2, respectively.

Supplementary figure 1: Screenshot of the registration of the chain of events leading to death in the paper certificate (in Norwegian).

Supplementary figure 2: Screenshot of the registration of the chain of events leading to death in the paper certificate (in Norwegian).

**Section 1.3 Information on current electronic certification system**

If the physician enters an ill-defined code (as defined by WHO; see Supplementary Table 2) as the only code on the certificate, a warning message appears in the next step of the electronic certification process. The pop-up states: “Could you help improve the death certificate reporting for us?”

It is followed by a more specific message, here using R99 (Ill-defined and unknown cause of mortality) as an example: “The diagnosis R99 – 'Ill-defined and unknown cause of mortality' is, on its own, an imprecise cause-of-death diagnosis. If possible, please include additional underlying conditions.” (Note: Authors own translation.)

Supplementary table 2: List of underlying causes of death that are considered garbage codes and creates a warning to the certifying doctor in the electronic system in Norway

| **ICD-10 code** | I46.1, I46.9, I50.9, N19, I99, J96.0, J96.9  R00-R57.1  R57.8-R59.9  R65.2-R65.3  R68.0-R94  R96-R99 |
| --- | --- |

**Section 2: Additional results**

Supplementary table 3: Crude and adjusted relative risk (RR) of garbage code use by type of death certificate, Norway 2018-2023.

|  |  | **Crude RR (95% CI)**  **Model 1** | **Adjusted RR^1^ (95% CI)**  **Model 2** | **Adjusted RR^2^ (95% CI)**  **Model 3** |
| --- | --- | --- | --- | --- |
| **Type of death certificate** | Paper | 1.00 (ref.) | 1.00 (ref.) | 1.00 (ref.) |
|  | Electronic | 0.79 (0.77-0.80) | 0.79 (0.78-0.80) | 0.77 (0.76-0.78) |
| **Sex** | Male | 0.87 (0.85-0.88) | 0.97 (0.95-0.98) | 0.98 (0.96-0.99) |
|  | Female | 1.00 (ref.) | 1.00 (ref.) | 1.00 (ref.) |
| **Age at death** | 0 | 0.12 (0.07-0.22) | 0.12 (0.06-0.22) | 0.11 (0.06-0.21) |
|  | 1-19 | 0.96 (0.81-1.14) | 0.92 (0.77-1.10) | 0.85 (0.71-1.02) |
|  | 20-49 | 1.60 (1.52-1.67) | 1.45 (1.38-1.52) | 1.36 (1.29-1.43) |
|  | 50-79 | 1.00 (ref.) | 1.00 (ref.) | 1.00 (ref.) |
|  | 80-89 | 1.58 (1.55-1.62) | 1.66 (1.62-1.70) | 1.74 (1.70-1.78) |
|  | 90+ | 2.24 (2.19-2.28) | 2.36 (2.31-2.42) | 2.46 (2.41-2.52) |
| **Place of death** | At home | 1.40 (1.37-1.44) | 1.48 (1.44-1.52) | 1.35 (1.31-1.39) |
|  | Hospital | 1.00 (ref.) | 1.00 (ref.) | 1.00 (ref.) |
|  | Nursing home | 1.27 (1.24-1.29) | 1.02 (1.00-1.04) | 0.98 (0.96-1.00) |
|  | Other known | 1.16 (1.10-1.22) | 1.18 (1.12-1.24) | 1.05 (0.99-1.11) |
| **Autopsy** | No autopsy | 1.00 (ref.) | NA | 1.00 (ref.) |
|  | Non-forensic | 0.61 (0.57-0.65) | NA | 0.89 (0.84-0.95) |
|  | Forensic | 1.04 (1.00-1.08) | NA | 1.26 (1.21-1.32) |
| **Centrality^a^** | Low | 1.13 (1.09-1.15) | NA | 1.03 (1.00-1.06) |
|  | Medium | 1.04 (1.02-1.06) | NA | 1.00 (0.98-1.02) |
|  | High | 1.00 (ref.) | NA | 1.00 (ref.) |
| **Additional information requested** | No | 1.00 (ref.) | NA | 1.00 (ref.) |
|  | Yes | 1.49 (1.42-1.55) | NA | 1.98 (1.89-2.06) |
| **Number of diagnoses** |  | 0.94 (0.94-0.95) | NA | 0.94 (0.93-0.94) |
| ^1^Adjusted for age at death, sex and place of death. ^2^Adjusted for age at death, sex, place of death, autopsy, centrality index, number of diagnoses on form and whether additional information had been requested by the registry. NA=Not applicable | | | | |

Supplementary table 4: List of the five most common major (class 1-2) and minor (class 3-4) garbage codes, by type of certificate

| **Diagnostic code** |  |  |  |
| --- | --- | --- | --- |
| **Paper certificates (N=112 396)** | **N** | **Percent of all deaths** | **Percent of GC in group (major or minor GC)** |
| **Major GC** | **16 280** | **14.5** | **100.0** |
| I50 Heart failure | 3273 | 2.9 | 20.1 |
| R96 Other sudden death, cause unknown | 1605 | 1.4 | 9.9 |
| X59 Accidental exposure to unspecified factor | 1542 | 1.4 | 9.5 |
| B99 Other and unspecified infectious diseases | 1058 | 0.9 | 6.5 |
| R54 Senility | 972 | 0.9 | 6.0 |
| **Minor GC** | **12 540** | **11.2** | **100.0** |
| J18 Pneumonia, organism unspecified | 3605 | 3.2 | 28.7 |
| I64 Stroke, not specified as hemorrhage or infarction | 2333 | 2.1 | 18.6 |
| C80 Malignant neoplasm without specification of site | 1067 | 0.9 | 8.5 |
| E14 Unspecified diabetes mellitus | 841 | 0.7 | 6.7 |
| I51 Complications and ill-defined descriptions of heart disease | 717 | 0.6 | 5.7 |
|  |  |  |  |
| **Electronic certificates (N=135 829)** | **N** | **Percent of all deaths** | **Percent of GC in group (major or minor GC)** |
| **Major GC** | **16 963** | **12.5** | **100.0** |
| I50 Heart failure | 3301 | 2.4 | 19.4 |
| R99 Ill-defined and unknown cause of mortality | 1140 | 0.8 | 6.7 |
| R96 Other sudden death, cause unknown | 1120 | 0.8 | 6.6 |
| R54 Senility | 1094 | 0.8 | 6.4 |
| X59 Accidental exposure to unspecified factor | 984 | 0.7 | 5.8 |
| **Minor GC** | **10 381** | **7.6** | **100.0** |
| J15 Bacterial pneumonia | 2111 | 1.6 | 20.3 |
| I64 Stroke, not specified as hemorrhage or infarction | 1204 | 0.8 | 11.6 |
| C80 Malignant neoplasm without specification of site | 1016 | 0.7 | 9.8 |
| J18 Pneumonia, organism unspecified | 955 | 0.7 | 9.2 |
| I69 Sequelae of cerebrovascular disease | 937 | 0.7 | 9.0 |

Supplementary figure 3: Proportion of deaths coded with a garbage code by class and year, 2018-2023.

Supplementary figure 4: Predicted proportion of garbage codes by form of certificate and age group when not classifying X40-X44 as garbage codes, adjusting for age of death, sex, and place of death, with interaction term between age at death and year of death, when setting place of death and sex equal to the reference group (“Hospital” and “Female”) to simplify the graphical representation.

Supplementary figure 5: Descriptive trends, measured as number of deaths reported with a specific code, in the top 10 major (left panel) and minor (right panel) garbage codes by type of certificate and year, Norway 2018-2023

References

1.	Naghavi M, Ong KL, Aali A, Ababneh HS, Abate YH, Abbafati C, et al. Global burden of 288 causes of death and life expectancy decomposition in 204 countries and territories and 811 subnational locations, 1990-2021: a systematic analysis for the Global Burden of Disease Study 2021. The Lancet. 2024;403(10440):2100-32.
